# Design and validation of a German version of the GSRS-IBS - an analysis of its psychometric quality and factorial structure

**DOI:** 10.1186/s12876-017-0684-8

**Published:** 2017-12-04

**Authors:** Sarah K. Schäfer, Kathrin Julia Weidner, Jorge Hoppner, Nicolas Becker, Dana Friedrich, Caroline S. Stokes, Frank Lammert, Volker Köllner

**Affiliations:** 1 Department of Psychology, Saarland University, Saarbrücken, Germany; 2 University Mannheim, I. Medical Clinic-Cardiology, Pneumology and Angiology Mannheim, Mannheim, Germany; 3 University Heidelberg, Clinic for Diagnostic and Interventional Radiology Heidelberg, Heidelberg, Germany; 4 Saarland University, Department of Medicine II – Gastroenterology und Endocrinology, Homburg, Germany; 5 Department of Psychosomatic Medicine, Rehabilitation Clinic Seehof, Lichterfelder Allee 55, 14513 Teltow, Germany; 6 Psychosomatic Rehabilitation Research Group, Department of Psychosomatic Medicine, Center for Internal Medicine and Dermatology Charité – Universitätsmedizin Berlin, Berlin, Germany

**Keywords:** Irritable bowel syndrome, Questionnaire, Questionnaire design, Self-report, Colonic diseases

## Abstract

**Background:**

Currently, a suitable questionnaire in German language is not available to monitor the progression and evaluate the severity of irritable bowel syndrome (IBS). Therefore, this study aimed to translate the Gastrointestinal Symptom Rating Scale for Irritable Bowel Syndrome (GSRS-IBS) into German and to evaluate its psychometric qualities and factorial structure.

**Methods:**

This study is based on a total sample of 372 participants [62.6% female, mean age = 41 years (SD = 17 years)]. 17.5% of the participants had a diagnosis of IBS, 19.9% were receiving treatment for chronic inflammatory bowel disease, 12.1% of the participants were recruited from a psychosomatic clinic, and 50.5% belonged to a control group. All participants completed the German version of GSRS-IBS (called Reizdarm-Fragebogen, RDF), as well as the Gießen Subjective Complaints List (GBB-24) and the Hospital Anxiety and Depression Scale - German version (HADS-D).

**Results:**

The internal consistency of the RDF total scale was at least satisfactory in all subsamples (Cronbach’s Alpha between .77 and .92), and for all subscales (Cronbach’s Alpha between .79 and .91). The item difficulties (between .25 and .73) and the item-total correlations (between .48 and .83) were equally satisfactory. Principal axis analysis revealed a four-factorial structure of the RDF items, which mainly resembled the structure of the English original. Convergent validity was established based on substantial and significant correlations with the stomach-complaint scale of the GBB-24 (*r* = .71; *p* < .01) and the anxiety (*r* = .42; *p* < .01) and depression scales (*r* = .43; *p* < .01) of the HADS-D.

**Conclusion:**

The German version of the GSRS-IBS RDF proves to be an effective, reliable, and valid questionnaire for the assessment of symptom severity in IBS, which can be used in clinical practice as well as in clinical studies.

**Electronic supplementary material:**

The online version of this article (10.1186/s12876-017-0684-8) contains supplementary material, which is available to authorized users.

## Background

Irritable bowel syndrome (IBS) is diagnosed on the basis of recurrent abdominal pain related to defecation or changes in stool frequency or form [1, 2]. The current prevalence of IBS ranges from 2.5 to 25%, depending on the diagnostic criteria used [3, 4]. For instance, the Manning criteria are associated with considerably higher prevalence rates when compared to the Rome I-III criteria. The prevalence of IBS is higher in women than in men. However, these gender differences decrease with increasing age [5, 6].

Patients with IBS experience a great degree of stress as a result of the condition. Several investigations with heterogeneous patient samples have illustrated that quality of life is perceived as being significantly impaired [7–10]. Further, adolescents suffering from IBS report a lower quality of life [11]. Moreover, IBS patients show considerably reduced health-related quality of life on all scales of the Short Form Health Survey (SF-36) [12] compared to other patient groups such as those with heart insufficiency [13]. A recent cross-sectional study from Norway has shown that IBS is associated with poor outcomes, particularly in the presence of health complaints, organic diseases, and affective disorders [14].

Three validated scales are available to assess IBS severity, of which the IBS severity scoring system (IBS-SSS) is most frequently used [15]. However, the IBS-SSS is an external assessment tool which has to be administered by the treating physician.

Häuser [16] has produced a German translation of the Rome III criteria for IBS that allows for a categorical decision about the presence of IBS. However, none of these tools include a patient self-assessment which would provide valuable additional information to the external assessment, particularly with regard to the disruptions in quality of life frequently occuring [9, 17]. The validated Gastrointestinal Symptom Rating Scale for Irritable Bowel Syndrome (GSRS-IBS) by Wiklund et al. [18] firstly represents such a diagnostic tool, which is currently only available in the English language.

The instrument was first introduced in 2003 with the aim to establish a self-assessment tool specifically adapted to IBS patients [18]. The GSRS-IBS is based on the Gastrointestinal Symptom Rating Scale (GSRS-IBS, [19]) developed for IBS patients and the Health-Related Quality of Life Questionnaire (HRQL, [20]) which is disease independent.

Currently, there is no appropriate questionnaire available in the German language (spoken by approximately 100 million people) to assess self-perceived symptom severity in IBS. However, such an assessment tool would be useful in the context of clinical practice as it would aid formation of a diagnosis and the monitoring of the course of IBS and additionally, for clinical studies.

### Objectives

Therefore, the aim of this study was to develop a German version of the GSRS-IBS (in the following called Reizdarm-Fragebogen, RDF) and to carry out a cross-cultural validation. For this purpose, the original questionnaire was translated into German and, in accordance with Wiklund et al. [18], was subsequently validated in a sample of IBS patients. Moreover, control groups were included to evaluate the specificity of the questionnaire.

## Methods

The purpose of the following section is to provide a brief overview of the translation and design process of the German version of GSRS-IBS and to describe its validation in a sample of different subgroups.

### Design of the German instrument

#### GSRS-IBS

The English version GSRS-IBS includes 13 items, which are rated on a seven-point scale ranging from "1 = no discomfort at all" to "7 = very severe discomfort". All items exclusively capture IBS constructs. The questionnaire was neither designed to produce ceiling effects nor to assess redundant information. A factor-analytical examination of all items in a validation cohort provided the following five factors: pain, diarrhoea, satiety, constipation, and bloating. Based on these findings identically named subscales containing two to four items were defined and demonstrated satisfactory internal consistencies reflected in Cronbach’s Alpha (α) ranging from .74 to .85.

#### Translation

In order to ensure a high quality translation process, a series of standards exists specifically for the cross-cultural translation of psychological and medical questionnaires [21]. The translation of the GSRS-IBS into German (RDF) was conducted in accordance with these recommended procedures. A preliminary version of the questionnaire was subsequently tested in a clinical sample. Contrary to the recommended standard specifications, no committee of experts was consulted concerning the quality of the questionnaire. Nonetheless, experienced gastroenterologists (including FL) confirmed its validity.

With regard to the specific process, two translators possessing the necessary knowledge of English and medical expertise independently translated the original questionnaire into their native German language. Both translations were then compared and the language was fine-tuned, which resulted in the translators agreeing on one version of the questionnaire. In order to assure the quality of the translation, the new version was retranslated back to English by two native English speakers who were not familiar with the original text. Except for some different choice of wording, the original English text did not differ from the retranslated version of the questionnaire, thus confirming the accuracy of the initial translation of the GSRS-IBS into German. Subsequently, the newly generated version of the questionnaire was given to a group of patients in a psychosomatic rehabilitation clinic in Blieskastel (MediClin Biestal-Kliniken), who were asked to check the comprehensibility of the items. None of these patients reported problems with comprehension. Therefore the German version was considered ready for implementation in the following validation study.

### Study process

#### Sample recruitment

##### Validation cohorts

Consecutive recruitment of the entire sample of 372 participants took place from April 2011 until June 2014. Out of these, 65 patients with a diagnosis of IBS were recruited from the department of Internal Medicine II at Saarland University Medical Centre in Homburg, Germany, and from an Internal Medicine private practice in Neunkirchen, Germany. For quality control purposes, the Rome III-criteria was used to validate the IBS diagnosis in these patients, and only those with an appropriate diagnosis were included. A random selection of IBS patients was not possible due to difficulties in ensuring a sufficient sample size during recruitment. 45 patients with a psychosomatic disorder were recruited from the MediClin Bliestal clinic in Blieskastel, Germany. Further, 74 patients with chronic inflammatory bowel disease (IBD) who were treated at Saarland University Medical Centre, Homburg, Germany, took part. A control group of students and orthopaedic patients with no gastroenterological conditions was also included in the study. The students voluntarily completed the questionnaires during an introductory course of the elective course ‘Medical case history’ at Saarland University, and the orthopaedic patients were questioned in a radiology practice in Neustadt/Weinstraße, Germany, whilst awaiting an MRI scan. The mean age of the included subjects was *M* = 41 years (*SD* = 17 years) (for sample characteristics see Table 1). The mean age would be considerably higher, if the students were excluded from the sample.

**Table 1:** Age and gender distribution of the entire sample

subsample	*n*	age (years)		women (percent)
		*M*	*SD*	
IBS patients	65	49	12	58.5
Chronic IBD patients	74	45	15	58.1
Psychosomatic patients	45	54	8	68.9
Orthopaedic patients and students	188	33	16	64.4
Total	372	41	17	62.6

*IBD* inflammatory bowel disease, *IBS* irritable bowel syndrome

#### Study procedure and instruments

##### Study procedure

In addition to the RDF, a German version of the Hospital Anxiety and Depression Scale (HADS-D) [22] as well as a short version of the Gießen Subjective Complaints List (GBB-24) [23] were used to verify convergent validity.

##### GBB-24

The GBB-24 assesses the psychosomatic causes of physical complaints [23]. The 24-item short version was given to subjects instead of the longer 57-item long version [24]. GBB-24 assesses organ-specific, objective and subjective symptoms. The following physical complaints were documented: cardiac and gastric complaints, pain in the limbs, and fatigue. The total score represents the overall subjective complaints [25]. The split-half-reliabilities were situated in a satisfactory range between *r* = .75 (gastric discomfort) and *r* = .94 (general complaints, total score).

##### HADS-D

The HADS-D questionnaire contains 14 items and assesses symptoms of anxiety and depression (7 items per scale) based on somatic and physical complaints [22]. The HADS-D can therefore be applied as a screening tool as well as to assess the severity of anxiety and depressive symptoms. Both, the anxiety and the depression scale showed a good internal consistency of α = .80.

### Statistical analyses

Initially, according to Wiklund et al. [18] a total score of the RDF as well as the individual sub-scores for the five proposed subscales in the English version were calculated for each subject by summing the total of the 13 items. Moreover, sum scores were calculated for the identified subscales in the German version following the outcome of a subsequent factor analysis. Regarding both, the GBB-24 and the HADS-D, scoring was carried out according to the respective manual instructions. A total score as well as scores for the proposed subscales were calculated for the GBB-24. Additionally, a total score for anxiety and depression symptoms was calculated for the HADS-D. In case of missing data for a maximum of two of the 13 RDF items, the missing values were replaced by the subject's mean score. If three or more answers were missing, the subject was excluded from all analyses. The same principle was applied to the HADS-D and the GBB-24. However, in case of the latter a subject was excluded from analyses if five items or more were missing.

A new variable was created to compare presence versus absence of IBS. Thereafter, a binary logistic regression was calculated in order to verify if the IBS patients could be distinguished from the other groups based on their RDF results.

Internal consistencies (Cronbach’s Alpha, α, [26]) were calculated for the entire questionnaire and for the subscales identified in the German version of the questionnaire, to determine the reliability of the RDF. Furthermore, the item difficulties and the item-total correlations were evaluated in order to assess item quality. To identify the factorial structure of the questionnaire, in comparison to the English validation, a slightly different procedure was employed: An exploratory principal axis analysis was performed with an oblique (Direct oblimin, Delta = 0) rotation. This analysis was chosen since exploratory factor analyses only consider item variance which is shared by at least two items. This was deemed to be more appropriate regarding measurement error [27]. Additionally, an oblique rotation appears to be more reasonable concerning the nature of IBS symptoms with respect to the chosen orthogonal rotation from Wiklund et al. [18]. To assume that those who suffer from severe constipation perceive the same amount of pain as those who do not suffer from constipation is implausible, which is why it seems appropriate to permit intercorrelations between factors.

The factor analyses were performed for the entire sample as well as separately for the subgroups (IBS, IBD, psychosomatic patients and control groups). Consequently, there is a factorial structure analysis for the entire sample (*n* = 372) from which 83% did not have a diagnosis of IBS. This can subsequently be compared to the factorial structure analysis in 65 patients with IBS. Moreover, this analysis in the IBS subsample can be compared with the IBS sample that was recruited for the validation of the original English version of the questionnaire. However, this comparison is limited due to the different types of factor analysis. Correlations with the GBB-24, its subscales and the HADS-scores were calculated to illustrate the questionnaire's convergent construct validity.

## Results

### Descriptive statistics

**Fig. 1:**
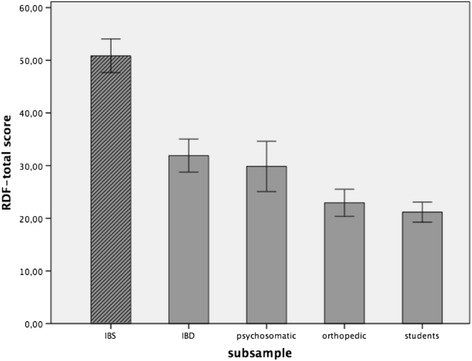
Means of the GSRS-IBS/RDF total score for the different subgroups. Legend: The bars indicate the 95% confidence interval of the group mean. *GSRS-IBS* Gastrointestinal Symptom Rating Scale for Irritable Bowel Syndrome; *IBD* (chronic) inflammatory bowel disease; *IBS* irritable bowel syndrome; *RDF* Reizdarm-Fragebogen

A comparison of the mean RDF scores between the different subgroups (see Table 2) revealed significant differences: The group of IBS patients had a significantly higher total score [*t*(111) = −12.27, *p* < .01], corresponding to more severe symptoms related to IBS (see Fig. 1). Likewise, the student [*t*(367) = 9.85, *p* < .01] and orthopaedic control groups [*t*(367) = 2.65, *p* = .004] differed significantly from the mean scores of all clinical samples. These considerable differences could be demonstrated for all subscales of the questionnaire, irrespective of whether they were based on the German or English factor structure. Binary logistic regression analysis was used to differentiate IBS patients from the rest of the cohort (non-IBS patients) using the RDF items. Overall, 90.3% of IBS cases were correctly classified [*χ* ^*2*^(13) = 140.95, *p* < .01].

**Table 2:** Mean values and standard deviations (in brackets) of the RDF scales for the various samples

Subgroups	Total (13 Items)	Pain (2)	Diarrhoea (4)	Constipation (2)	Satiety (2)	Bloating (3)
IBS	50.85 (12.86)	8.03 (2.92)	14.74 (5.93)	5.74 (3.83)	7.89 (3.80)	14.45 (4.60)
Chronic IBD	31.90 (13.57)	4.59 (2.85)	11.62 (6.01)	3.30 (2.20)	4.41 (2.76)	7.98 (4.55)
Psycho-somatic	29.85 (15.90)	3.86 (2.35)	8.54 (5.17)	4.36 (3.24)	4.38 (3.13)	8.72 (5.68)
Control group	21.84 (10.59)	3.61 (2.43)	6.10 (3.26)	2.85 (1.86)	3.44 (2.06)	5.84 (3.90)
Total	29.88 (16.18)	4.61 (3.05)	9.00 (5.75)	3.63 (2.77)	4.52 (3.14)	8.12 (5.37)

*IBD* inflammatory bowel disease, *IBS* irritable bowel syndrome

### Assessment of item quality

#### Item quality

The item difficulties of the RDF ranged between *p* = .25 and *p* = .73 in the entire sample (see Table 3). As expected they were slightly lower (*p* = .41 to *p* = .72) in the IBS sample and had a lower range. The majority of the items were medium in difficulty with a few being very difficult or very easy. Such a distribution appears to be reasonable for differentiating IBS patients. The mean item-scale correlation for the entire sample was *r* = .65. This corrected item-scale correlation is satisfactory. However, it was considerably lower for the IBS patients (mean *r* = .40). That is, however, to be evaluated in context to the later described diverging factorial structure.

**Table 3:** Item difficulties and item-total correlations for RDF in the entire sample and in the group of IBS patients

	IBS patients		Entire sample	
	Item difficulties	Item-total correlations	Item difficulties	Item-total correlations
1. Stomach pain	.62	.37	.36	.65
2. Relief through bowel movement	.52	.50	.30	.75
3. Passing gas	.71	.42	.41	.77
4. Stomach gas	.72	.68	.40	.83
5. Constipation	.41	.30	.25	.51
6. Diarrhoea or frequent bowel movement	.48	.18	.30	.57
7. Liquid stool	.41	.21	.73	.50
8. Hard stool	.41	.30	.26	.48
9. Urge to empty bowel	.59	.36	.37	.65
10. Feeling of complete emptying of the bowel	.62	.56	.34	.75
11. Feeling of fullness after meals	.55	.42	.31	.62
12. Prolonged feeling of fullness	.58	.39	.33	.65
13. Bloated stomach	.64	.51	.35	.77

*IBS* irritable bowel syndrome, *RDF* Reizdarm-Fragebogen

Equally satisfactory are the results of the reliability analyses, with a Cronbach’s Alpha in the entire sample of α = .92 for the RDF total scale (α = .77 in the IBS sample), and Cronbach’s Alpha for the subscales of the German version within an acceptable range from α = .84 to α = .93 (α = .72 to α = .92 in the IBS sample) [28].

### Factor analyses

The suitability of conducting a factorial analysis on the data derived from the entire sample was reflected in a KMO value (Kaiser-Meyer-Olkin measure of sample adequacy) of KMO = .88 and a significant Barlett test [*χ* ^*2*^(78) = 3457.68, *p* < .01]. The principal axis analysis with oblique rotation (Oblimin direct, Delta = 0) provided a two-factorial result for the overall sample according to Kaiser-Guttmann (Table 4). The first factor indicated an eigenvalue of 6.69 while the second factor lagged noticeably behind with an eigenvalue of 1.77. The KMO value of .68 for the IBS patients was not as strong, but nevertheless still appropriate for conducting a factor-analytical evaluation. The lower value might only reflect a significantly reduced sample size. The Barlett test of sphericity was also significant [*χ* ^*2*^(78) = 463.37, *p* < .01]. The principal axis analysis with an identical rotation provided a four-factorial structure for the IBS sample according to Kaiser-Guttman. The first factor is characterized principally by bloating-related sensations and the passing of gas, whereas the second factor mainly summarises symptoms of diarrhoea. The third factor is associated with constipation while the fourth factor is related to the urge to empty the bowel and the ensuing relief that the patients feel. In total, 64.5% of the item variance could be explained by these four extracted factors.

**Table 4:** Loading matrix (pattern matrix) of the oblique rotation (factor eigenvalue in brackets)

	Factor 1 Bloated stomach (2.48)	Factor 2 Diarrhoea (2.35)	Factor 3 Constipation (2.80)	Factor 4 Pain and feeling of tension (2.52)
1. Stomach pain	−.04	.06	.07	**.49**
2. Relief through bowel movement	.10	−.13	−.13	**.83**
3. Passing gas	**−.93**	.05	−.14	−.01
4. Stomach gas	**.87**	.08	.18	.05
5. Constipation	.06	−.39	**.44**	.12
6. Diarrhoea or frequent stool	.11	**.91**	.02	.09
7. Liquid stool	.05	**.83**	.10	.09
8. Hard stool	.03	−.40	**.45**	.13
9. Urge to empty bowel	−.05	.28	−.10	**.62**
10. Feeling of complete emptiness of the bowel	.13	−.09	.14	**.56**
11. Feeling of fullness after meals	−.12	.13	**.92**	−.01
12. Prolonged feeling of fullness	.03	−.03	**.85**	−.09
13. Bloated stomach	.23	.03	**.49**	.10

*Note*: Factor loadings > .40 in bold print

### Intercorrelation of subscales

To determine the correlations between the various questionnaires, for RDF a total score with item 3 recoded was calculated. This process takes the negative sign of the factor scores into account. Subsequently, bivariate correlation analyses were conducted between the subscale scores and the total score of the RDF, both for the entire sample and separately for patients with IBS (Table 5). This analysis revealed that the first subscale, ‘bloating’ (items 3 and 4), is the most independent. However, in IBS patients, a substantial association with constipation-related complaints was observed. Concerning the subscale 'diarrhoea' (items 6 and 7), significant correlations were shown for 'constipation' however not for IBS patients), 'pain and feelings of tension'. Significant correlations were identified between the subscale constipation (items 5, 8, 11, 12, and 13), and all other subscales. However, the correlations with 'diarrhoea' and 'pain and feelings of tension' are not significant for IBS patients. The subscales 'pain and feelings of tension' are considerably associated with the scales 'diarrhoea' and 'constipation' (but not for IBS patients). However, they were not correlated with 'bloating' in both, the IBS and the total sample. All subscales correlated significantly with the RDF total score (with the exception of the ‘bloating’ subscale in the entire sample).

**Table 5:** Correlations and internal consistencies of the various dimensions in the entire sample (first correlation, *n* = 372) and the subsample of IBS patients (second correlation, *n* = 65)

	1	2	3	4	5
Bloating (1)	.93/.90	.01/.05	.13*/.32*	−.01/.07	.04/.25*
Diarrhoea (2)		.89/.92	.37**/−.11	.58**/.31*	.64**/.36**
Constipation (3)			.85/.82	.63**/.22	.82**/.64**
Pain and feeling of tension (4)				.84/.72	.91**/−.75**
RDF total (5)					.92./.77

The diagonal contains internal consistencies of each subscale, first for the entire sample and second for the IBS subsample

*IBS* irritable bowel syndrome, *RDF* Reizdarm-Fragebogen

*p < .05

**p < .01

### Convergent validity

The correlations between the HADS-D and GBB-24 total scores and RDF scores reflect the convergent validity for the latter (Table 6). All analysed samples showed significant and similar correlations between the responses on the RDF and those on the subscale for stomach pain in the GBB-24. As expected, the correlation was particulary strong in IBS patients (*r* = .65, *p* < .01). The same applies to the correlation of the GBB-total value that is associated with general abdominal pain and the total scores of the RDF. Correlations were significant for the entire sample (*r* = .56, *p* < .01) as well as for the group of IBS patients (*r* = .44, *p* < .01). Likewise, the correlations for the subscales of the HADS-D depression and anxiety were significant for the entire sample. It is, however, apparent that the correlations of RDF score and HADS scores are notably lower in the group of IBS patients than in the total sample.

**Table 6:** Correlations of RDF total scores and other instruments for the total sample (first correlation, *n* = 372) and the sample of IBS patients (second correlation, *n* = 65)

	1	2	3	4
RDF total (1)	–	.42**/.15	.26**/.12	.26**/.56**
HADS-D Anxiety (2)		–	.49**/.71**	.23**/.20
HADS-D Depression (3)			–	.55**/.12
GBB-24 stomach discomfort (4)				–

*GBB-24* Gießen Subjective Complaints List 24, *HADS-D* Hospital Anxiety and Depression Scale - German Version, *IBS* irritable bowel syndrome, *RDF* Reizdarm-Fragebogen

*p < .05

**p < .01

## Discussion

The results suggest that the RDF as the German version of the GSRS-IBS, can be used as a reliable and valid questionnaire to assess self-perceived severity and to monitor progress in IBS patients in German-speaking countries. The present analyses establish the RDF as a reliable and economical tool. This is evidenced by the sufficient psychometric quality of the tests, its plausible, factorial structure as well as its selective sensitivity for IBS symptoms. In line with this, significant differences in the total RDF score were reported for IBS patients in comparison to other clinical and non-clinical samples. Its suitability is further reflected by the convergent validity apparent in the correlations between the RDF and the GBB-24 and the HADS-D. The present study further demonstrates that the German version of the RDF is also suitable for subjective severity assessment of IBS and that the same IBS construct is consequently acquired in the English-speaking world as well as in Germany [29].

However, it must be noted that the factorial structure of the German version does not perfectly match the original English version. The method of choice to prove the similarity of the factorial structure would certainly have to be a confirmatory factor analysis [30]. This method was deliberately not applied in the present study due to methodological reasons. A different exploratory approach was chosen that, on the one hand, seems to be more appropriate for the data - with respect to the inter-factor correlations and the considered variances - but, on the other hand, distinctly limits the comparison of the findings with the results from Wiklund et al. [18]. The employed factorial analysis resulted in four instead of five factors and raises concerns over whether the five-factorial structure found in the English original and the associated interpretation of the scales should be adjusted. It may be advisable to initially focus on the value of the entire RDF and to subsequently define the exact scale structure and compile indications of its interpretation, only after a further validation sample.

Despite the scale structure that still needs to be investigated, the RDF can certainly be recommended for its application in a screening procedure. However, in order to ensure a meaningful implementation in daily clinical practice or in general practitioner practices, it would be reasonable to define cut-off-values. This would require a larger sample of IBS patients, which would also be necessary to determine the extent to which the questionnaire is suitable to assess the subjective and additionally the objective degree of IBS. A comparison with the currently used severity scores in daily clinical practice would be particularly important. Such a comparison would also establish to what extent the RDF can be meaningfully implemented to document the (subjective) progression of IBS and if it should be included in the standard therapy plan.

### Limitations

On a critical note, only 65 patients with IBS were recruited for this investigation, a small sample compared to the 234 IBS patients used in the validation of the original English version. This relatively small sample size is especially associated with limitations in the interpretation of the factorial analytical results. Smaller samples (and thus samples with a greater range restriction) are more likely to be associated with a larger number of factors, which could present an alternative explanation for the diverse factorial structures in the total cohort and in the group of IBS patients. The fact that the IBS patients were less suitable for the factor analytic investigation is reflected in the significantly lower KMO coefficients, even when compared to the entire sample. Moreover, the control group comprising students is a further limitation, as characteristics such as age, years of education and socioeconomic status differed in the two groups, thus limiting their comparability. However, the inclusion of orthopaedic patients should have reduced such influences. Nevertheless, a replication and expansion of this study with a larger patient cohort and a matched control group would be desirable.

Additionally, not all aspects of the test quality could be taken in consideration. For instance, findings regarding stability of the results in terms of retest-reliability are lacking. The coherence of the HADS-D and the GBB-24 point towards a persuasive convergent construct validity, while a proof of a sufficient discriminant validity is indeed missing. Likewise, it seems reasonable to examine the extent to which the RDF scores correlate with other relevant variables within IBS.

## Conclusion

The German version of the GSRS-IBS RDF proves to be an effective, reliable, and valid questionnaire for the assessment of self-perceived symptom severity in IBS, which can be used in clinical practice as well as in clinical studies.

## Additional files

Additional file 1: German**_**Questionnaire_Reizdarmfragebogen. German version of the GSRS-IBS/Reizdarmfragebogen including scoring instructions. (PDF 54 kb)

Additional file 2: Data_Validation_Reizdarmfragebogen. Dataset used during this study. (XLSX 103 kb)

## Abbreviations

GBB-24: Gießen Subjective Complaints List Gießener Beschwerdebogen 24
GSRS-IBS: Gastrointestinal Symptom Rating Scale for Irritable Bowel Syndrome
HADS-D: Hospital Anxiety and Depression Scale - Deutsche Version (German Version) HRQL Health-Related Quality of Life Questionnaire
IBD: (Chronic) inflammatory bowel disease
IBS: Irritable bowel syndrome
KMO: Kaiser-Meyer-Olkin measure of sample adequacy
RDF: Reizdarm-Fragebogen
