# Supplementary material for: Design and validation of a German version of the GSRS-IBS - an analysis of its psychometric quality and factorial structure
Source: BMC Gastroenterol. 2017 Dec 4;17:139. doi: 10.1186/s12876-017-0684-8 (PMC5715554; doi:10.1186/s12876-017-0684-8)
Supplement: Supplementary file 1 — German_Questionnaire_Reizdarmfragebogen. German version of the GSRS-IBS/Reizdarmfragebogen including scoring instructions. (PDF 54 kb) [file 12876_2017_684_MOESM1_ESM.pdf]

REIZDARM-FRAGEBOGEN

German Version of the GSRS-IBS

INSTUKTION:

Bitte entscheiden Sie sich auf einer Skala von 1 bis 7 wie die folgenden Fragen auf Sie zutreffen wobei [Skalenbeschreibung]

SCALE:

1 = überhaupt keine Beschwerden

2 = geringfügige Beschwerden

3 = leichte Beschwerden

4 = mäßige Beschwerden

5 = mäßig starke Beschwerden

6 = starke Beschwerden

7 = sehr starke Beschwerden

ITEMS:

1. Litten Sie in der vergangenen Woche unter Bauchschmerzen?
2. Litten Sie in der vergangenen Woche unter Schmerzen oder Beschwerden im Bauchraum, die sich nach Stuhlgang lösten?
3. Litten Sie in der vergangenen Woche unter abgehenden Blähungen?
4. Litten Sie in der vergangenen Woche unter Blähungsgefühl?
5. Litten Sie in der vergangenen Woche unter Verstopfung?
6. Litten Sie in der vergangenen Woche unter Durchfall (häufiger Stuhlgang)?
7. Litten Sie in der vergangenen Woche unter flüssigem Stuhlgang?
8. Litten Sie in der vergangenen Woche unter hartem Stuhlgang?
9. Litten Sie in der vergangenen Woche unter dem dringenden Bedürfnis, zur Toilette zu müssen, um Ihren Darm zu entleeren?
10. Litten sie in der vergangenen Woche unter dem Gefühl, dass Ihr Darm nach dem Stuhlgang nicht vollständig entleert war?
11. Litten Sie in der vergangenen Woche unter Völlegefühl, kurz nachdem Sie mit der Mahlzeit begonnen hatten?
12. Litten Sie in der vergangenen Woche unter Völlegefühl, das noch lange nach dem Essen anhielt?
13. Litten sie in der vergangenen Woche unter einem sichtbar aufgeblähten Bauch?

Subscales in the German version of the GSRS-IBS/Subskalen des Reizdarm-Fragebogens:

Bloating: Items 3 (recoded) and 4/ **Völlegefühl**: Items 3 (recodiert) und 4

Diarrhoea: Items 6 and 7/ **Diarrhoe**: Items 6 und 7

Constipation: Items 5, 8, 11, 12 and 13/ **Verstopfung**: Items 5, 8, 11, 12 und 13

Pain and feeling of tension: Items 1, 2, 9 and 10/ **Schmerzen und Spannungsgefühl**: Items 1, 2, 9 und 10
